# Acrylamide and Advanced Glycation End Products in Frying Food: Formation, Effects, and Harmfulness

**DOI:** 10.3390/foods14193313

**Published:** 2025-09-24

**Authors:** Arslan Rasool, Xiaoyu Luo, Qiqi Zhang, Caihua Jia, Siming Zhao, Ru Liu, Jianhua Rong, Guangsheng Zhou, Bo Wang, Jie Kuai, Jing Wang, Jie Zhao

**Affiliations:** 1 MOE Key Laboratory of Environmental Food Science, College of Food Science and Technology, Huazhong Agricultural University, Wuhan 430070, China; arslanrasool437@gmail.com (A.R.); 18717137132@163.com (X.L.); qiqizhang@webmail.hzau.edu.cn (Q.Z.); zsmjx@mail.hzau.edu.cn (S.Z.); liuru@mail.hzau.edu.cn (R.L.); rong@mail.hzau.edu.cn (J.R.); 2 MOA Key Laboratory of Crop Ecophysiology and Farming System in the Middle Reaches of the Yangtze River, College of Plant Science and Technology, Huazhong Agricultural University, Wuhan 430070, China; zhougs@mail.hzau.edu.cn (G.Z.); wangbo@mail.hzau.edu.cn (B.W.); kuaijie@mail.hzau.edu.cn (J.K.); wangjing@mail.hzau.edu.cn (J.W.); zhaojie@mail.hzau.edu.cn (J.Z.)

**Keywords:** food frying, acrylamide, advanced glycation end products, pathways, the Maillard reaction, food safety

## Abstract

Frying food can provide an attractive flavor relatively quickly; however, it inevitably produces some safety risks during high-temperature processing, with potentially adverse human health effects. Prolonged exposure to high temperatures during frying might raise the concentration of some harmful compounds that accumulate from the complex chemical reactions taking place inside the food matrix. This review elaborates on the development of food risk factors during frying, adding acrylamide (AA) and advanced glycation end products (AGEs), which are involved in various health problems, including chronic illnesses and carcinogenesis. The two commonly recognized pathways for acrylamide formation include the Maillard reaction pathway and the acrylic acid pathway, with the Maillard reaction considered to be the primary pathway for AGE formation. The processing conditions and food components that affect the formation of these toxic compounds are then specified, demonstrating the importance of factors including type of oil, composition of food (such as moisture and fat content), frying temperature, and duration. Finally, the corresponding health hazards posed by the risk factors are summarized, with an emphasis on the long-term effects of acrylamide and AGE exposure on human health. Increased risks of neurotoxicity, cancer, inflammation, and metabolic diseases have been associated with both compounds. The aim is to clarify the formation pathways, influencing factors, and health impacts of risk factors in frying food and to provide a reference for the prevention of food safety problems caused by acrylamide and advanced glycation end products.

## 1. Introduction

Frying is a common processing technique that quickly provides a desirable color and flavor to food and is widely used in food preparation. During frying, simultaneous mass and heat transfer occur, with heat and oil entering the food while moisture exits. Foods are usually cooked thoroughly within 0.5 to 5 min at high frying temperatures [1]. Frying at high temperatures can cause various food safety hazards, including acrylamide (AA) [2], advanced glycation end products (AGEs) [3], 5-hydroxymethylfurfural [4], and polycyclic aromatic hydrocarbons [5], among others. Acrylamide is often formed in carbohydrate-rich foods when reducing sugars (glucose, fructose, etc.) react with amino acids (particularly asparagine) by means of the Maillard reaction [6]. This reaction involves the interaction between electrophilic carbonyl groups from reducing sugars with nucleophilic amino groups of proteins, peptides, or amino acids, leading to the formation of acrylamide through the Maillard reaction. It can also create reactive carbonyl compounds, which can induce carbonyl stress and eventually promote the formation of AGEs [7]. Acrylamide in fried foods may be toxic to animals and humans [5]. Research on acrylamide has mainly focused on starchy foods treated at high temperatures, like French fries and bread [8]. Asparagine and reducing sugars serve as the key reaction substrates for acrylamide in starchy foods. Asparagine, the primary amino acid precursor, plays an important role in the formation of AA, which is directly affected by the presence of mono- and disaccharides, including fructose, sucrose, and glucose.

The length of a sugar’s chain affects its reactivity; shorter carbon chains result in higher AA production. Pentoses participate more readily in the Maillard reaction and contribute to stronger browning, thus making them generally more reactive than hexoses and disaccharides. These two substances exist simultaneously; substances with a lower content are the key factors restricting acrylamide formation [9]. Few published studies have examined the effects of new processing methods on the mechanisms of production of AGEs, even though heating effects on AGE levels in foods are well established. Non-thermal processes use short treatment times and avoid high temperatures. In comparison to conventional thermal processing, non-thermal methods cause less alteration of sensory qualities, and the nutritional components are better preserved. This implies that AGE formation is reduced under non-thermal processing. Most of the new thermal technologies enable rapid heating, which helps to shorten processing time and, as a result, lower rates of AGE formation [10]. The presence of AGEs may disrupt glucose metabolism in the body and increase the risk of inflammation, damage to endothelial and renal function, and major chronic diseases [11]. A series of complex non-enzymatic reactions occur between free amino groups of proteins, peptides, or amino acids and the carbonyl groups of reducing sugars such as glucose and fructose, leading to the formation of AGEs [12]. Food processing procedures, conditions, and composition strongly influence the formation of these hazardous compounds, with the Maillard reaction, lipid oxidation, and protein oxidation being key pathways during frying [2]. AGEs are generally classified into two groups: exogenous AGEs, which originate from external sources like smoking, consumption of AGE-rich foods, and exposure to ultraviolet (UV) light, and endogenous AGEs, which are produced in the body under physiological conditions. Long-lived proteins, including collagen, are particularly prone to glycation, leading to tissue stiffening and functional decline with age. Key AGEs, including CML, pentosidine, and MG-H1, along with reactive dicarbonyl compounds like methylglyoxal, are widely used as biochemical markers of in vivo glycation. Notably, dicarbonyls serve as highly reactive intermediates that act as AGE precursors, both in food systems and within the human body [13], thereby linking dietary exposure to pathological processes, such as cardiovascular disease, diabetes, and neurodegeneration. Current research on AGEs has mainly focused on dairy products, beef, chicken, and other foods [14]. The AGE content in protein foods is higher due to the abundant amino groups essential for glycation reactions, whereas the acrylamide content is lower [15]. AGEs are particularly important because they are linked to aging-related effects, cardiovascular diseases, and chronic diseases like diabetes. Food processing parameters, specifically time and temperature, have a significant effect on AGEs and acrylamide in foods. To reduce their formation in food, it is important to understand how they interact with different food matrices. The formation mechanisms, significant variables, and possible health risks of toxic chemicals formed during high-temperature processing and frying are examined in this review. After studying acrylamide and AGEs separately, this review aims to provide a comparative overview of their mechanisms of formation, key influencing factors, and mitigation strategies. Their formation is closely linked via common cooking processes and chemical pathways such as lipid oxidation. Such a comparative platform offers a means for developing simultaneous reduction strategies in fried foods and is an important advancement over discussing these toxicants in isolation.

## 2. Formation of Risk Factors

### 2.1. Formation of Acrylamide

Processing food at high temperatures leads to the formation of acrylamide (AA) (C3H5NO; prop-2-enamide), a substance recognized for its carcinogenic, immunotoxic, neurotoxic, and reproductive toxicity [8]. However, few studies have investigated acrylamide in foods fried at high temperatures, leaving a gap in understanding how it is generated under common frying conditions. According to Delgado-Andrade, Mesías, and Morales [16], acrylamide formation can vary significantly depending on a number of variables, including moisture content during frying, the food matrix structure, and products of frying oil degradation. These factors affect the concentration of precursors, including asparagine and reducing sugars.

**Figure 1:**
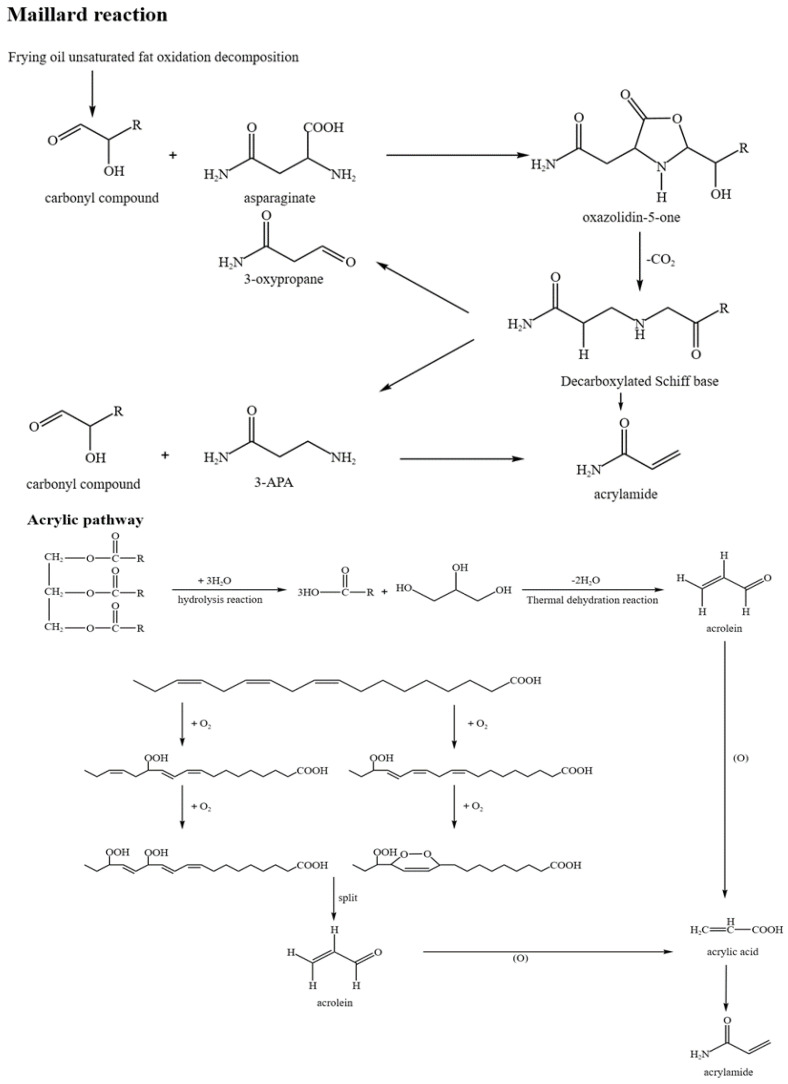
Maillard reaction and acrylic acid pathway for the formation of acrylamide.

The two recognized pathways to produce acrylamide are the Maillard reaction pathway and the acrylic acid pathway, as shown in Figure 1. The most common pathway, the Maillard reaction, initiates with condensation between the amino acid asparagine and reducing sugar (e.g., glucose) to form a Schiff base. This Schiff base is subsequently decarboxylated to form the key intermediate, 3-aminopropionamide (3-APA). Degradation of 3-APA by deamination or direct hydrolysis represents the direct precursor of acrylamide formation [17]. Additionally, acrylamide can be produced through asparagine, acrylic acid, nitrogenous compound self-reaction, and the asparagine pyrolysis pathway [18]. In starchy foods, acrylamide is normally produced through the Maillard reaction between the amino acid asparagine and glucose, fructose, or reducing sugars. When asparagine is heated alone, a small amount of acrylamide is directly formed by decarboxylation and deamination [19]. Pre-treatment strategies, such as enzymatic removal of asparagine or modification of frying conditions, can significantly reduce acrylamide formation without compromising food quality. Furthermore, acrylamide formation influences the oxidative stability of frying oils, as advanced lipid oxidation products increase the level of reactive carbonyl compounds, which in turn promote acrylamide synthesis [20].

Under high-temperature treatment, the amino group of asparagine and the carbonyl group of reducing sugar undergo dehydration and condensation to form a Schiff base, followed by decarboxylation. An intramolecular rearrangement to acrylamide formation or dehydration occurs to form 3-aminopropionamide, followed by deamination to acrylamide [21]. In addition to reducing sugars in food, oil oxidation can generate carbonyl groups [22]. For example, carbonyl groups are mainly generated through fat oxidation in protein-rich foods. Controlling oil quality during repeated frying cycles is critical to reducing the formation of reactive carbonyl compounds and subsequent acrylamide production.

The acrylic acid pathway induces acrylamide formation via the reaction of free ammonia with acrolein or acrylic acid under high-temperature conditions [23]. This pathway proceeds by a nucleophilic addition–elimination mechanism. Acrylic acid is activated either by forming a reactive ester or by dehydrative activation. The amino nucleophile then attacks the electrophilic carbon of the activated acrylic acid, leading to the removal of water molecules and formation of acrylamide molecules [24]. Acrolein and acrylic acid can originate from a wide range of sources, including non-enzymatic degradation of monosaccharides due to heating, decomposition of fats and oils, amino acids and proteins during high-temperature heating, and degradation. For example, the degradation of aspartic acid, serine, and cysteine in food can produce acrylic acid through intermediates such as lactic acid. Furthermore, heating alanine and arginine above 180 °C can also lead to acrylic acid formation [25]. The acrylic acid pathway occurs more widely in foods but is limited by free ammonia. This indicates that the concentration of free ammonia is a key determinant of acrylamide formation. A relatively high reaction temperature is required for this reaction to be effective, which is why the acrylamide produced via the acrylic acid pathway is generally lower than that formed through the asparagine pathway. Table 1 shows the occurrence of acrylamide in different foods, along with their respective cooking methods, acrylamide content (μg/kg), detection methods, and risk categories. In the case of potato tubers, Schouten [26] conducted several pretreatment experiments prior to frying to investigate their potential capacity to reduce acrylamide formation. Treatment was designed to either decrease the contents of acrylamide precursors or alter the microstructure of the tubers to limit the acrylamide formation when cooked. Specifically, W (dipping in water) promotes the leaching of reducing sugars and asparagine into the soaking medium; PW (pulse electric field in water followed by dipping in water) enhances cell membrane permeability, thus promoting release and removing precursors; and Y (dipping in yeast aqueous suspension) utilizes yeast metabolism to consume reducing sugars and amino acids, thereby preventing acrylamide formation. It is important to note that these pretreatments were applied only to potato tubers and are not to be taken to other foods listed in Table 1. This highlights how acrylamide is influenced by the food type and processing conditions that mitigate exposure through industrial strategies.

### 2.2. Formation of AGEs

A variety of AGEs with diverse structural characteristics have been identified, including Nε-(carboxymethyl)-lysine (CML), N-(carboxyethyl)-lysine (CEL), and other AGEs. CML was the first AGE to be isolated and detected from glycosylated proteins [33]. AGEs are generally classified into two groups: exogenous and endogenous. Endogenous AGEs are produced in the body from excess proteins and sugars during non-enzymatic glycosylation within organs, tissues, and body fluids. Exogenous AGEs mainly enter the human body by the ingestion of foods with dietary AGEs (dAGEs) being the primary source [34]. Animal foods rich in proteins and lipids produce more AGEs during cooking. The oxidation of fats and proteins produces dicarbonyl compounds, which further promote AGE formation [35]. Meat products rich in fat and protein show higher AGE levels than other types of food [36]. Additionally, cooking methods involving high temperatures and low moisture for prolonged periods, such as frying, are known to promote AGE formation. Multiple studies have indicated that AGEs are closely related to chronic inflammation, degenerative diseases, aging, insulin resistance, tumor development, and other disorders [37].

**Figure 2:**
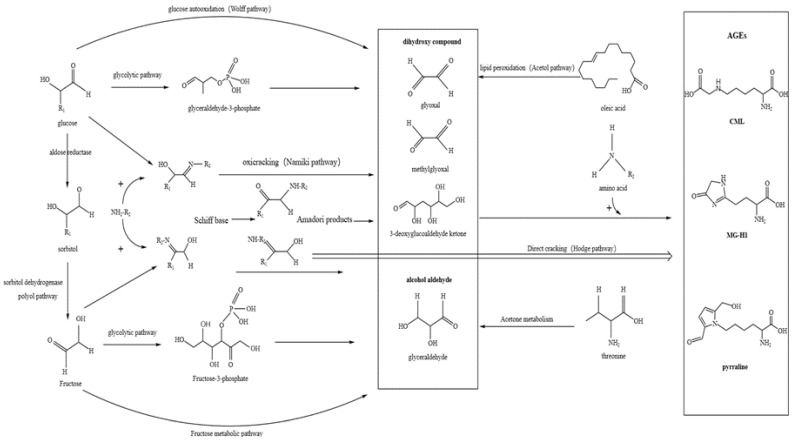
Formation of advanced glycation end products.

The AGE formation pathway is shown in Figure 2. The Maillard reaction is widely recognized as the primary pathway for AGE formation [38] and can be divided into three distinct stages [11]. In the first stage, carbonyl compounds react reversibly with amino compounds to form unstable Schiff base adducts, which are later converted into more stable Amadori rearrangement products (ARPs). During the intermediate stage, some Amadori products undergo direct oxidation and pyrolysis to form AGEs. Meanwhile, other reactions, influenced by factors including pH and temperature, lead to the formation of highly reactive dicarbonyl compounds—key precursors of AGEs—like glyoxal (GO), methylglyoxal (MGO), and 3-deoxyglucosone (3-DG)—through processes including dehydration, oxidation, and rearrangement. Finally, these AGE precursors react with amino groups such as the guanidine group of arginine and the free amino group of lysine, resulting in the formation of stable and irreversible AGEs [14]. An essential intermediate in AGE formation, active dicarbonyl compounds can also be formed through lipid peroxidation via the acetol pathway [22], Schiff base oxidative cracking (Namiki pathway), the polyol pathway, amino acid-derived ketone metabolism [39], and glucose autoxidation [40], in addition to Amadori product rearrangement.

AGEs are primarily present in protein-rich fried meat products. Studies have demonstrated that oil oxidation can accelerate AGE formation due to the interaction between proteins and lipid oxidation products, resulting in high levels of Schiff base adduct [41]. Active free radicals are produced during oil oxidation, and electrons are transferred to reaction intermediates to promote further oxidation, which also significantly promotes the Maillard reaction, increasing the production of AGEs [18]. At the same time, carbonyl compounds, including glyoxal (GO) and methylglyoxal (MGO), formed by oil oxidation, react with the protein-free amino groups, acting as carbonyl donors to produce more AGEs [42]. Additionally, the types of carbonyl and amino compounds play a significant role in influencing the end products of the Maillard reaction. Amino acids react more easily than macromolecular proteins. Free amino groups of proteins are the main sites for carbonyl-amine condensation, in which lysine (primary amine) and arginine residues (secondary amine) are the most commonly glycosylated amino acids [11]. Primary amines react more positively than secondary amines, which describes why the glycosylation products of lysine residues in food typically exhibit the highest AGE content, making them useful as markers for AGEs. Include carboxymethyl lysine and carboxyethyl lysine [38]. Yu [43] explored the effects of lipid oxidation on the formation of Nε-carboxymethyllysine and Nε-carboxyethyllysine during storage. Results express a significant correlation among fluorescent AGEs, carboxymethyllysine carboxyethyllysine, and MG-H1, as well as both fat and malondialdehyde content (*p* < 0.01). The Maillard reaction and lipid were found to cooperatively influence CML production in Chinese sausages, with lipid oxidation playing a significant role in AGE progression. AGEs, mainly CML, occur through glycation and oxidative reactions during food processing and physiological metabolism. These compounds contribute to the development of chronic diseases like diabetes, atherosclerosis, or neurodegenerative disorders. Table 2 shows the level of common AGEs found in food products, categorized by preparation methods, glycation markers, and analytical detection method. This also helps in understanding the cooking techniques and food composition effects on AGE accumulation.

## 3. Effects on the Formation of Risk Factors and Inhibition Methods

### 3.1. Effects on the Formation of Acrylamide in Food

**Figure 3:**
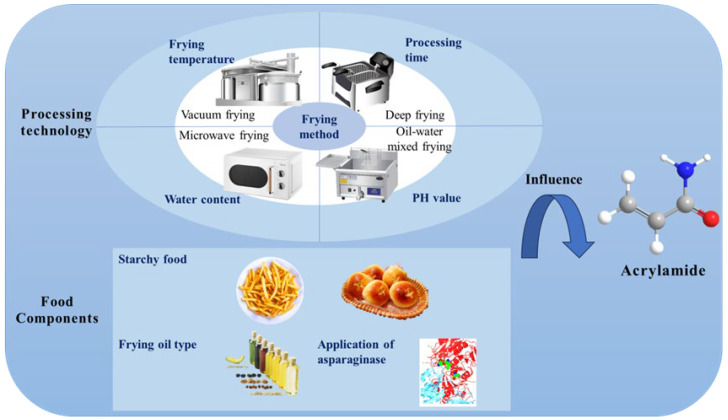
Influences on the formation of acrylamide in food.

During heating, asparagine reacts with reducing sugars through dehydration to form a Schiff base by N-glycosylation and decarboxylation. This initiates a cascade of reactions, including hydrolysis, decarboxylation, and deamination, which generate reactive intermediate compounds. These intermediates ultimately lead to acrylamide formation [46]. Due to its potential carcinogenicity in humans, acrylamide formation has been associated with various frying techniques. Since its discovery in 2002, acrylamide has attracted remarkable interest in the fields of food science and environmental research. Currently, research on acrylamide mainly focuses on starch-rich foods, like French fries and potato chips. The processing temperature, time, frying method, food composition, and water content significantly affect acrylamide formation, as shown in Figure 3. Among the food ingredients, asparagine and reducing sugars play a key role in this process [47].

#### 3.1.1. Processing Conditions

Frying temperature and duration are key factors influencing acrylamide formation. Acrylamide formation typically begins when the temperature exceeds 120 °C. The nucleophilic addition reaction between reducing sugars and asparagine requires sufficiently high heat to proceed [48]. In the asparagine-reducing sugar model, acrylamide content rises as the temperature increases from 120 °C to 170 °C but begins to decrease when the temperature exceeds 170 °C. Some studies have shown that acrylamide content decreases significantly above 200 °C [49]. Water activity also plays an important role. Acrylamide formation decreases in high-moisture environments and is not observed when water activity exceeds 0.8. As water activity decreases, acrylamide formation increases, reaching its maximum level when the water activity is approximately 0.4 [50].

Acrylamide formation is also influenced by pH changes. At lower pH values, the amino group of asparagine is protonated and loses its ability to attack the carbonyl group, thus preventing Schiff base formation. Lowering the pH of the system can reduce acrylamide production without affecting the taste or color of food [51]. Acidic pretreatments have been shown to effectively reduce acrylamide. For example, Liu [52] showed that soaking the raw potato slices in an acetic acid solution (1.0%, 2.5%, or 5.0%) for 8 h reduced glycoside alkaloids by more than 50% and decreased acrylamide level by over 95%. Higher temperatures and longer soaking times may negatively affect organoleptic properties such as taste and texture. Frying methods also vary in their effects on acrylamide formation. Sansano [53] found that compared with conventional methods, microwave and deep frying achieved acrylamide reductions of approximately 37% and 83%, respectively. Air frying also reduces acrylamide content by 47% to 90%, depending on cooking parameters. Vacuum frying proves even more effective, achieving reductions of 80% to 88% [54]. Both air and vacuum frying are efficient, while close comparison reveals a key trade-off: vacuum frying is better at preserving heat-sensitive nutrients and colors due to the absence of oxygen, while air frying is more convenient and cost-effective to implement at home. The use of technology therefore depends on specific nutritional and economic goals [55]. These modern frying technologies not only lower acrylamide content but also preserve product quality, offering healthier alternatives to traditional methods.

#### 3.1.2. Food Composition

Food composition is also an important factor affecting the formation of acrylamide, with asparagine being the primary route in fried foods. Research indicates that foods higher in reducing sugars and asparagine tend to have elevated acrylamide levels. In starchy foods, where reducing sugars are abundant, free asparagine is the key factor influencing acrylamide formation. Conversely, for protein-rich foods, such as certain aquatic products naturally high in asparagine, the availability of reducing sugars serves as the limiting factor for acrylamide production. Y. Yang, Achaerandio, and Pujola [56] stated that in French fries, the formation of acrylamide occurs through the Maillard reaction between asparagine and reducing sugars. It was also found that the acrylamide levels in fresh potatoes were correlated with the content of reducing sugars and sucrose. The type and condition of oil used also affect acrylamide formation because carbonyl compounds involved in the Maillard reaction are also produced by oil degradation. Continuous use of frying oil significantly increases acrylamide levels. Moreover, oil types matter: hazelnut oil exhibits lower acrylamide content than corn oil, while olive oil shows intermediate levels [23].

The diversity of raw materials, particularly potato varieties, also affects acrylamide formation. Y. Yang, Achaerandio, and Pujola [56] studied three potato varieties to examine the relationship between acrylamide precursors in fresh tubers and acrylamide formation in fried potatoes. Acrylamide level was positively correlated with sucrose and reducing sugar contents in fresh potatoes. In Agria potatoes, the acrylamide formation primarily resulted from sucrose hydrolysis because of their low reducing sugar content, as shown in Figure 3. Furthermore, L-asparaginase acts as an effective acrylamide-reducing agent by catalyzing the hydrolysis of the asparagine amide side chain, producing ammonia and aspartic acid. Since aspartic acid cannot serve as an acrylamide precursor, this reaction reduced acrylamide formation. Recent studies have focused on optimizing L-asparaginase application for industrial food processing. For instance, immobilization of the enzyme on nanoparticles or in edible films allows reuse and prevents inactivation during frying, improving cost-effectiveness and efficiency. These advances demonstrate significant improvements over simple dipping procedures, shifting the focus towards practical, large-scale applications [57]. Application of L-asparaginase effectively inhibited acrylamide formation in fried potato chips [58], without affecting color or taste. First isolated from *Aspergillus terreus* [59], the enzyme reduced the acrylamide concentration to 815.63 μg/kg at the optimal concentration of 4 U/mL. Although asparaginase significantly reduced acrylamide formation by targeting asparagine, the resulting levels remained slightly above the EFSA benchmark of 750 μg/kg for fried potato products. Importantly, the treatment did not significantly alter the reducing sugar content of potato slices, thereby preserving their natural quality [57].

### 3.2. Methods to Hinder the Acrylamide Formation in Food

Several strategies have been applied to reduce acrylamide formation in foods by limiting the Maillard reaction. Acrylamide formation can be controlled in three ways: modifying raw materials, optimizing processing conditions, and adding exogenous additives [19]. Since the asparagine pathway is the primary route for acrylamide formation in fried food, controlling asparagine and carbonyl sources in raw materials is crucial for effectively mitigating acrylamide production. In cereals, the asparagine levels directly determine the amount of acrylamide formation under high-temperature treatment due to the abundant reducing sugars. Asparagine is more abundant in aquatic products, and it is hypothesized that reducing sugar content may be the main factor limiting the asparagine pathway.

Optimizing processing conditions can significantly reduce acrylamide production. Thermal methods, such as UV irradiation and microwave heating, along with non-thermal techniques like UHP, homogenization, or PEF, have been shown to effectively reduce acrylamide formation in foods [60]. Additionally, prolonged bread fermentation and acidification of cookies significantly inhibited acrylamide production [51].

The addition of exogenous substances to foods is a widely adopted strategy to mitigate acrylamide formation. For instance, in a simulated asparagine–glucose model system, the addition of buckwheat extract significantly decreased acrylamide levels [19]. Cereal-based products comprise a significant portion of the daily diet. Common cereal-derived foods like bread, breakfast cereals, cookies, crackers, cereal-based baby food, and biscuits contain varying levels of acrylamide, ranging between 38 and 407 μg/kg on average and ranging from 60 to 1600 μg/kg among the top 5% of samples examined [61]. To reduce the acrylamide levels in foods, the European Union has set benchmark values and implemented mitigation measures [32].

The addition of antioxidants reacts with the carbonyl group produced by the oxidation of fats and oils, thus preventing the acrolein pathway [19]. Babu [62] found that succulent cactus extract was effective in preventing the onset of lipid oxidation, thereby reducing acrylamide production and mitigating the oxidative deterioration of its own antioxidants in potatoes. However, the use of exogenous substances is more restricted due to their potential to diminish the sensory quality of food. It is important to implement policies based on the type and processing stage of the product, ensuring its final nutritional value, organoleptic properties, or safety. Each strategy should also be carefully assessed for compatibility with the food business, costs, and regulatory compliance.

### 3.3. Effects on the AGE Formation in Food

**Figure 4:**
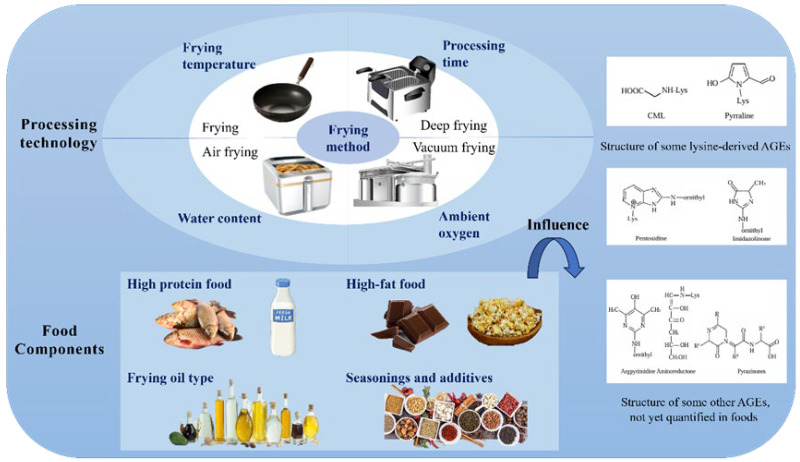
Influences on the formation of AGEs in food.

The Maillard reaction, a crucial chemical process between amino acids and reducing sugars, is the primary pathway for AGE formation in food. Consequently, the factors affecting the Maillard reaction, like heating temperature, duration, pH levels, and food composition, including moisture, fat, and protein content, play a critical role in determining AGE levels. Moreover, seasonings and additives commonly used in processed foods, such as spices, soy sauce, sugar, salt, and vinegar, further contribute to the modulation of AGE formation during cooking and food preparation, as shown in Figure 4.

#### 3.3.1. Factors Influencung AGE Formation

Both heating temperature and duration significantly influence the Maillard reaction. Different thermal processing methods, such as deep-frying and high-temperature frying, can increase AGE levels [14]. In particular, high storage temperature and ambient oxygen levels significantly affect the fluorescent AGE levels (*p* < 0.05), with CML concentrations increasing as temperature and storage duration rise (*p* < 0.05) [63]. G. Chen and Smith [45] reported on the content of AGE in pork, chicken, beef, and fish (salmon and tilapia) cooked by three ordinary cooking methods: deep-frying (204 °C), baking (177 °C), and barbecue (232 °C). All three methods could detect CML in the samples, but its level depended on the meat type, cooking conditions, and final internal temperature. CML content during frying and roasting at high temperatures was higher, and roast beef had the highest CML concentration (21.8 μg/g). P. Han [64] found that the frying method significantly influences AGE formation, which is positively correlated with temperature and amino groups and negatively correlated with water content. Qin [65] investigated AGE formation in deep-fried, fried, baked, and air-fried fish cakes. The results indicated that AGE content on the surface of the fish cake significantly increases with longer heating time and exhibits a negative correlation with water content and a positive correlation with oil content, oxidation products, and yellowness (b*). Additionally, compared to other frying methods, air-fried fish cakes result in lower levels of fats and AGEs while producing a color like deep-fried fish cakes.

#### 3.3.2. Food Components

Food composition, including the relative contents of proteins, fats, and sugars, is a key factor affecting AGE formation. Foods with high protein and fat content usually have higher AGE levels after high-temperature heating. As shown in Figure 4, foods rich in carbohydrates, such as starches, fruits, and vegetables, have lower AGE levels after high-temperature processing [66]. Meanwhile, foods high in fat, like chocolate and popcorn, tend to have higher AGE contents. In contrast, AGE levels in starchy foods made from dough are lower [67]. Furthermore, as fat peroxidation usually occurs in high-fat foods such as meat, lipid peroxidation can produce diketones, which participate in the Maillard reaction and promote AGE formation. Moisture content also plays a role in AGE production.

Lower water content accelerates the Maillard reaction, while higher water content significantly slows its rate, thereby reducing AGE formation. Paradoxically, although higher moisture during cooking inhibits formation, the final acrylamide concentration is often measured in foods that retain a moist interior. The minimum amount of acrylamide production in starch matrices has been observed at water concentrations between 25% and 40%. According to the data obtained from Ciesarová, Kiss, and Kolek [68], the acrylamide levels increased significantly beyond this range. Consequently, the primary factors influencing acrylamide production are the water content in food, as well as reaction time and temperature. AGE formation during processing is also affected by pH. Under acidic conditions, the free radical pathway of the Maillard reaction is relatively weak, leading to lower AGE formation. When pH increases to approximately 10, the free radical reaction is enhanced, resulting in a marked increase in AGE content.

### 3.4. Methods to Hinder the AGE Formation in Food

AGE formation during frying is affected by multiple factors. High temperature and prolonged heating time significantly increase AGE levels. High-temperature, low-moisture cooking methods like microwaving, roasting, and frying cause a more drastic rise in AGE levels [14]. In contrast, for cooking methods with high moisture and lower temperature, such as steaming and boiling, the Maillard reaction occurs slowly, resulting in low AGE levels compared to baking and frying [44]. Therefore, processing conditions, such as temperature, moisture content, and other parameters, should be regulated to avoid high temperature and low moisture conditions for prolonged heating. The CML levels formed after the steaming treatment of scallops were significantly lower than those of the frying and roasting treatments [38].

Antioxidants can inhibit lipid oxidation in foods, thereby reducing the accumulation of reactive carbonyl groups, which in turn decreases AGE formation. Given the Maillard reaction’s key role in flavor, taste, and color, measures to reduce or capture reactive carbonyl compounds (RCO) may be more effective in inhibiting AGE formation than directly restricting the Maillard reaction. Some antioxidants, such as aminoguanidine, are often used as carbonyl compound trapping agents. It has been shown that AGE production in heat-treated olive oil was completely inhibited by aminoguanidine and partially inhibited by dibutylhydroxytoluene (BHT) [15]. Aminoguanidine reduced AGE production by inhibiting the onset of glycosylation in foods and was more effective than BHT. However, aminoguanidine is not suitable for addition to food products due to its side effects and is more often used as a clinical therapeutic agent. Meanwhile, many natural antioxidants can be used as carbonyl trapping agents, including flavonoids and polyphenols. Khalifa [69] found that two anthocyanins extracted from Morus alba fruits reduced AGE levels by capturing RCO, especially GO. Quercetin, a natural flavanol, can also inhibit AGE formation by trapping RCO [70]. In addition, Chen [71] reported that phenolic acids had better inhibition of AGE formation in an oleic acid–bovine serum protein system, with dihydroferulic acid having the most significant effect.

The application of external breading or hydrophilic coatings can also be effective in inhibiting the penetration of frying oil into foods during the frying process and reducing moisture loss. Jiang [72] reported that fish pieces coated with an outer batter containing hydrophilic colloids exhibited a significantly lower AGE content than those directly fried. The application of fish paste coating is effective in preventing the penetration of fats and oils into the inner layers of the fish cake [73]. The effect of breadcrumbs and wheat flour on AGE levels is also of interest because of the frequent need to use external breading when frying fish pieces. Protein coatings or fiber-containing paste layers can effectively inhibit the absorption of fats and lipid oxidation of foods during frying [74]. However, it remains to be investigated whether the combined use of protein coatings and antioxidants can reduce AGE production in foods by maintaining high water content or inhibiting lipid oxidation.

## 4. The Harmfulness of Risk Factors in Fried Foods

### 4.1. Harmfulness of AA in Fried Food

#### 4.1.1. The Action Pathway of AA

Mass transfer in food frying includes oil absorption and water evaporation, in which oil absorption can increase the flavor and nutritional value of food. However, a long-term high-fat diet can pose significant health risks. Fried and baked starchy foods can produce acrylamide. Additionally, bread is the primary source of acrylamide in baked goods (accounting for about 11% of its content) and contributes to around 25% of consumers’ overall dietary intake [75,76]. Several factors, including fermentation, the composition of wheat flour, and baking conditions, might influence the amount of acrylamide in bread. Wheat flour contains around 374 mg/kg of free ASN and 8460 mg/kg of reducing sugar. As the main component in bread, it plays a primary role in acrylamide formation during baking operation [77]. This toxic and harmful ingredient was identified as a second-class carcinogen by the IARC and has teratogenic, carcinogenic, and neurotoxic properties [78]. Both IARC and JECFA have thoroughly conducted the toxicological effects of acrylamide and classified its associated health risk [79]. Due to its small molecular size and high biological activity, acrylamide can be absorbed actively or passively and easily enter the circulatory system through the skin and digestive tract, which is harmful to human health. In addition to dietary causes, acrylamide exposure can occur through tobacco use, inhalation, and skin absorption.

In many animal studies, acrylamide mutates genes in mammalian somatic and germ cells, causing tumors and even cancers in the brain, lungs, breast, uterus, and other organs [80]. However, its carcinogenic mode and mechanism of action in specific organs are still debated and require further research. Currently, the key to acrylamide (AA) carcinogenesis is the production of oxidative metabolites. Both acrylamide and epoxy acrylamide can inhibit cell mitosis or meiosis and can also cause abnormal hormone levels and deplete glutathione. Subsequently, the redox balance is destroyed, interfering with gene expression and leading to carcinogenesis.

#### 4.1.2. Acrylamide Toxicity

**Figure 5:**
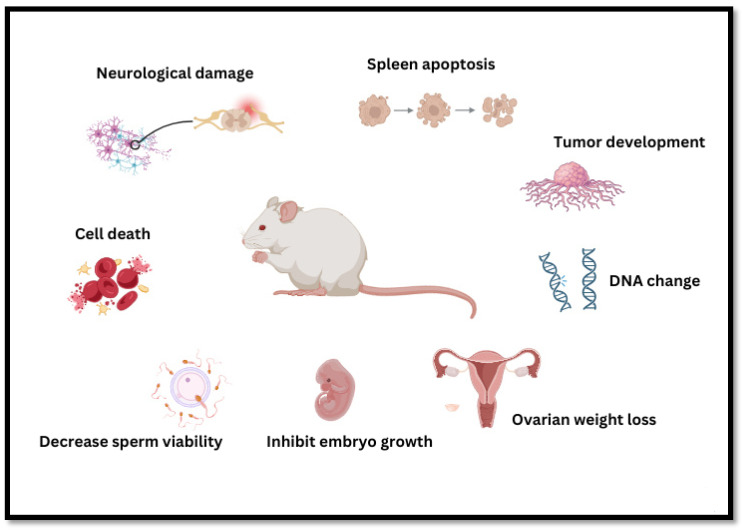
Summary of the adverse effects of acrylamide exposure in a murine model.

The toxic effects of acrylamide mainly impact neurological health, splenic apoptosis, tumor progression, DNA alteration, cell death, reproductive toxicity (reduced sperm viability, inhibition of embryonic growth, and reduction in ovarian weight), and overall systemic dysfunction, as shown in Figure 5.

##### Reproductive Toxicity

Acrylamide causes reproductive toxicity in animals. Prolonged exposure can irreversibly damage the reproductive system, including a decline in sperm viability, an elevation in the deformity rate, and a decrease in the testicular cell apoptosis rate in male animals. Additionally, by disrupting the mammalian target of rapamycin/phosphoinositide 3-kinase/protein kinase B signaling pathway and inducing apoptotic factors via mitochondrial dysfunction, acrylamide can cause cellular autophagy. This results in sperm malfunction, testicular tissue morphological damage, DNA damage, and ultimately cell death [81]. Female animals showed decreased ovarian weights, decreased estrogen secretion, and increased follicle apoptosis rates. Animal embryos also experience adverse pregnancy outcomes, such as increased abortion rate and fetal weight loss. Acrylamide can inhibit embryo growth, impair placental labyrinth angiogenesis, and downregulate placental development-related genes. In addition, acrylamide further increases the risk of kidney disease and reduces the head circumference of newborn fetuses. Pregnant females should minimize acrylamide exposure to protect fetal health. Aldawood [82] found that acrylamide induces ovarian dysfunction by affecting the release of steroids and increasing apoptosis. Idris [83] demonstrated that acrylamide exposure in pregnant Wistar rats is a significant risk factor for mothers and their offspring.

##### Neurotoxicity

Acrylamide poses serious risks to the nervous system in animals, potentially leading to neurological damage, limb weakness, and cognitive impairments. Preliminary studies suggest that damage to nerve endings in the CNS and PNS may be connected to AA-induced neurotoxicity. Acrylamide can modify β-tubulin, β-actin, and other cytoskeletal proteins, causing neurotoxicity by damaging the architecture of neurons [84]. Acrylamide exposure causes rat hippocampi to exhibit reduced levels of BDNF and hyperphosphorylation of Tau tubulin, which leads to synaptic loss and spatial cognitive impairment [85]. Acrylamide binds to the sulfhydryl groups of hemoglobin and tissue proteins. A small amount of acrylamide remains in the nerve tissue for more than 14 days, resulting in cell degeneration and partial neuropathy. Several studies have investigated behavioral changes and alterations in cerebellar morphology caused by chronic acrylamide exposure. Experimental studies have found that rats administered 5 mg/kg acrylamide exhibit lessened balance, reduced hindlimb muscle strength, and impaired gait and motor coordination. Separately, it has been shown that acrylamide can induce neuronal autophagosome accumulation, endoplasmic reticulum expansion, and mitochondrial swelling and increase the level of apoptosis to produce neurotoxicity [86]. L. Yang [87] studied the mechanism of mitochondrial damage caused by acrylamide and found that it triggers the aggregation of mitochondrial ROS, disrupts mitochondrial biogenesis and dynamics, causes mitochondrial DNA damage, and eventually leads to mitochondrial dysfunction and apoptosis. Additionally, since acrylamide is a food-borne hazard formed via the Maillard reaction in starchy foods cooked at high temperatures, a high-calorie diet can significantly aggravate acrylamide-induced neurotoxicity.

##### Immune Toxicity

Acrylamide exposure has toxic effects on immune organs and can significantly inhibit immune function, including pathological, cellular, and humoral immunity. Acrylamide is also an immunotoxin, harming the immune system and increasing disease risk. Numerous studies have demonstrated that acrylamide can lead to excessive apoptosis in splenic cells and thymocytes, resulting in a remarkable reduction in the ratio of whole blood T cells to NK cells, increased serum cytokine IFN-γ levels, and reduced lymphocyte and macrophage function. Some studies by Zamani [88] have found that acrylamide exposure can cause immune hemocytopenia, alterations in body and organ weights, and pathological changes in the spleen of mice. Moreover, acrylamide can indirectly damage the immune system by increasing lipid peroxides and reducing free radical scavengers’ activity.

Komoike, Nomura-Komoike, and Matsuoka [89] fed adult zebrafish a diet containing acrylamide for one month and analyzed the histology and pathology of the spleen. The results showed that ingesting acrylamide-containing food can cause splenic injury, including bleeding, cyst formation, and inflammation, accompanied by immune responses. This includes the upregulation of major inflammatory cytokines in the spleen, black macrophage centers, and macrophage activation. J. Fang and Liang Chun [90] evaluated the immunotoxicological effects of acrylamide in female BALB/c mice by assessing humoral immunity, cellular immunity, immunopathology, and nonspecific immunity. Acrylamide exposure has been revealed to reduce the spleen, body, and thymus weight, decrease lymphocyte count, and induce pathological changes in the spleen, lymph glands, and thymus. In addition, splenocyte proliferation and serum hemolysis (HC50) in response to mitogens like concanavalin A and cytokines in the high-dose acrylamide group were significantly inhibited, leading to immune dysfunction. Guo [91] investigated the association between acrylamide exposure and its metabolite, glycidylamide hemoglobin adduct, and their associations with allergy-related health outcomes in the general American population and found that these two substances can induce allergic reactions such as asthma, hay fever, itchy rash, and sneezing, which may be related to acrylamide-induced immunodeficiency. These studies suggest that acrylamide exposure may negatively affect the immune system function, potentially leading to immunotoxin effects; however, the full spectrum of its toxicity and the precise underlying mechanisms are not fully understood and require further investigation.

##### Carcinogenicity

Based on experimental studies, IARC assigned acrylamide as a probable human carcinogen (Group 2A) in 1994. Similarly, in 2011, the US National Toxicology Program’s report on carcinogens categorized it as “reasonably anticipated to be a human carcinogen”. Experimental studies suggest that acrylamide can cause cancer in humans by forming adducts with DNA and other biomolecules such as hemoglobin. This interaction leads to chromosomal abnormalities and genetic mutations, significantly increasing the probability of cancer development [92]. Acrylamide exposure may cause cellular gene mutations such as those observed in animal lungs [93] and disrupt metabolic processes like glycine metabolism. These effects can lead to tumors and monocytic leukemia in various body parts [94]. Some epidemiological studies have found that acrylamide also has a carcinogenic effect, and the intake of foods containing acrylamide can cause breast, endometrium, prostate, stomach, liver, and oral cancer [95].

Hogervorst [96] was the first to analyze the effect of gene–acrylamide interactions on ovarian cancer risk. Several significant relations were found between acrylamide consumption and single-nucleotide polymorphisms (SNPs) in the HSD3B1/B2 gene cluster, suggesting that acrylamide may contribute to ovarian cancer through sex hormone effects.

The carcinogenic mechanism of acrylamide remains unclear but is related to the epoxidation metabolite glycidamide (GA). GA is considered the primary direct carcinogenic agent of acrylamide in laboratory animals. Some studies by L. Zhang [97] demonstrated the oxidative damage caused by acrylamide and GA in vivo and preliminarily explored the possible carcinogenic mechanism. The strong oxidative ability of GA to attack DNA and regulate DNA repair-related genes is the key mechanism for its carcinogenicity in mouse liver. Both acrylamide and GA can significantly upregulate the oncogene Rad51 and epidermal growth factor receptor (EGFR). Furthermore, acrylamide downregulates the tumor suppressor gene p21 and activates inflammatory factors. These actions may collectively initiate cancer-related pathways such as PI3K and ErbB and induce tumorigenesis and cancer. Simultaneously, GA and many reactive oxygen species can directly attack DNA. Acrylamide and GA also regulate Rad51 and p21 genes to impair DNA repair, resulting in oxidative damage that can lead to cancer. Additionally, multiple studies have found no precisely significant correlation between dietary acrylamide intake and risk of various types of cancers [98], whereas some studies have reported an increased risk of kidney, endometrial, and ovarian cancers. However, inadequate exposure assessment may have allowed for misclassification or underestimation of acrylamide exposure, and more studies of acrylamide exposure assessment on improved diets will be conducted in the future as well.

### 4.2. Harmfulness of AGEs in Fried Food

Studies have reported that foods that have been processed at high temperatures, like grilling, frying, and baking, are particularly high in dAGEs found in foods, specifically those rich in fats and proteins, as well as in grilled or fried meat products. AGEs are also absorbed, broken down, and eliminated through the gastrointestinal tract upon consumption. This contributes to the elevated risk of chronic conditions, including diabetes, hypertension, atherosclerosis, and kidney disease [99], but also leads to increased exogenous AGEs in the body.

#### 4.2.1. The Action Pathway of AGEs

AGEs influence human health in two primary ways: (1) by cross-linking with proteins, which alters their structural and functional properties, causing dysfunction and disease, and (2) by interacting with specific receptors, triggering inflammation, oxidative stress, and adaptive immune responses through different cellular signals [100]. The first pathway connected to the accelerated production of endogenous AGEs is closely linked to an increased risk of diabetes [101]. Numerous studies revealed that the production of heterogeneous AGEs and precursors rises in a short duration and that glycation processes generally occur more quickly in heat-intensive food preparation than in vivo [102]. In the second pathway, common key receptors include RAGE (a member of the immunoglobulin family, which functions as a multiligand receptor), AGE-R1/OST-48, AGER2/80K-H, and other receptors. Exogenous AGEs accumulate in tissues and organs, influencing the expression and activation of RAGE. Once RAGE is activated, transcription factors such as NF-κB, STAT3, and HIF-1α are induced, accelerating cytokine secretion. As a result, this generates reactive oxygen species (ROS) and develops an inflammatory response, leading to more AGE formation, which conducts the circulation of inflammatory responses [103].

#### 4.2.2. AGEs and Chronic Diseases

**Figure 6:**
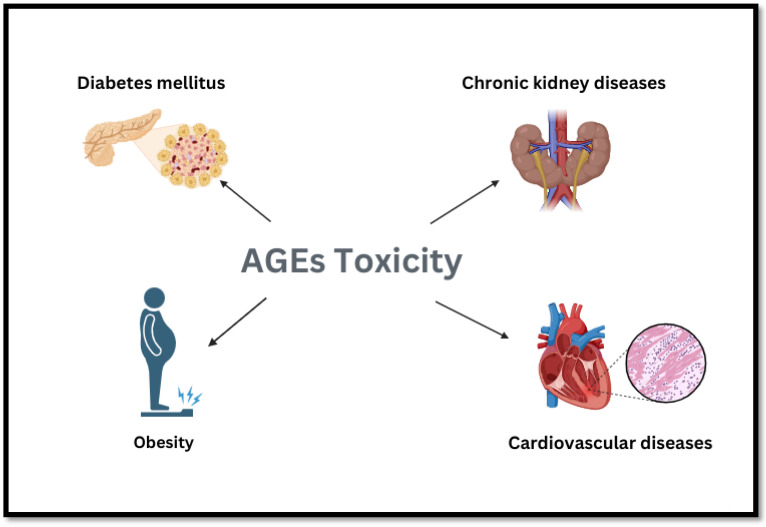
Overview of the toxic effects of AGEs.

The toxic effects of AGEs are generally associated with their role in the formation of diabetes, cardiovascular disease, CKD, and obesity, with an emphasis on their systemic effects on metabolism and organ health as shown in Figure 6.

##### Obesity

Studies have shown that adult obesity and overweight are closely related to the intake of highly processed food, such as high-temperature frying [104]. AGEs are by-products of food processing, and numerous studies have investigated their impact on obesity. Cordova [105] demonstrated a relationship between dietary intake of AGEs and adult changes in adult body weight. They found that dairy products, sugar, meat, cakes, cereals, and fish were the major contributors to AGE intake. In the case of milk products, Yajing Xie and van der Fels-Klerx [106] suggested that when dAGEs are formed, the nutritional value of the final product is reduced. It has been reported that over 20% of lysine residues in milk products go through lactosylation during glycation. Since infants cannot digest lactulosyllysine, glycation reduces the amount of lysine available in milk and decreases the digestibility of milk protein. Studies have shown that higher dAGE intake is associated with increased weight gain. Mirmiran [107] evaluated the connection between dAGEs and obesity. They found that eating more dAGEs increased the risk of abdominal obesity, which was still significant after adjusting for potential confounding factors, suggesting that dAGEs may be a link between the modern diet and obesity. Additionally, since the 1980s, the prevalence of chronic kidney disease (CKD) has increased by approximately 25%, affecting people of all ages. This may be partly due to changes in lifestyle and greater consumption of “Western” diets, which are usually energy-intensive, characterized by low fruit and vegetable consumption and excess intake of animal protein and heavily processed foods, and may lead to an increased intake of AGEs [108], triggering metabolic dysfunction, contributing to an increase in obesity and diabetes, and further promoting the productivity of endogenous AGEs in the body.

##### Diabetes Mellitus and Related Complications

AGEs play an important role in the progression of diabetes and its related complications. In diabetes patients, AGE formation is accelerated due to elevated circulating glucose levels, raised AGE precursors, increased oxidative stress, and slower protein turnover [109]. Dietary AGE intake will lead to aggravated diabetes. Foods rich in fat and protein contain high amounts of carboxymethyllysine, whereas carbohydrate-rich foods generally contain low levels. The dietary intake of AGEs is increasing, and food sources may exceed the endogenous AGE content. Limiting dietary AGEs can reduce serum AGE concentrations [110], and the amount of foodborne AGEs is sufficient to promote type 2 diabetes. Studies in humans and mice have shown diets rich in AGEs can increase AGE accumulation, including MG-H1, and in a non-diabetic subject, raise inflammatory and oxidative stress biomarkers associated with insulin resistance [111]. Additionally, insulin-resistant mice with type II diabetes were fed high- or low-AGE diets. After 20 weeks, the mice on the high-AGE diet showed an increase in the plasma AGE levels and body weight gain. Heidari [112] showed that AGE levels in the blood may be measured quantitatively and used as a biomarker for the length of diabetes. The lowest AGE concentrations were diagnosed in new patients, whereas the group with chronic disease had the highest levels of glycotoxins.

##### Cardiovascular Disease and Its Associated Problems

Over the past two decades, increasing evidence has shown that AGEs have been linked to the development of age-related chronic diseases like cardiovascular diseases. Glycosylation of proteins, peptides, and amino acids contributes to an increase in the level of collagen crosslinking in the vascular wall. This may explain why AGEs are intimately related to cardiovascular diseases and their complications. This phenomenon may lead to further deposition of glycosylated low-density lipoproteins on the vascular wall, and increased cross-linking can also lead to the dysfunction of macrophages, cell foam formation, and progression of atherosclerotic plaque formation over a long period [113]. Moreover, whole-mount in situ hybridization utilizing a variety of biomarkers showed acrylamide modifies the atrioventricular canal differentiation [114].

Connection between AGEs and endothelial cells reduces the endothelial barrier function and increases permeability, leading to the entry of subendothelial lipids. The interaction between AGEs and the receptor RAGE stimulates the expression of adhesion molecules and promotes monocyte migration across the endothelium, resulting in increased foam cell formation [113]. In vitro experiments using mouse aorta and endothelial cells have demonstrated that methyl acetaldehyde, a precursor of AGEs, decreases endothelial angiogenesis through peroxynitrite dependence by involving RAGE-mediated and autophagy-induced degradation of VEGFR2. This process can also influence vasodilation and endothelial cell proliferation and migration.

### 4.3. Health Implications of Acrylamide–AGEs Interactions

Although acrylamide and AGEs are often studied separately, both share a common source of dietary exposure through the consumption of fried foods. More importantly, their health impacts are not isolated but have synergistic effects, which increase overall toxicity. One critical mechanism of synergy is the enhancement of oxidative stress and inflammation. Acrylamide decreases glutathione (GSH) [84], the master antioxidant, leaving the body’s first line of defense system compromised. This causes a pro-oxidant environment that enhances the ability of AGEs to develop ROS and initiate inflammatory pathways via the RAGE receptors [103]. This cycle of oxidative stress and inflammation contributes to the development of chronic diseases such as neurodegeneration, diabetes, and cardiovascular disease. Furthermore, both compounds have a capacity to induce cellular damage through protein adduction—acrylamide to neurofilaments [84] and AGEs to collagen and plasma proteins [113]. This mechanism can lead to an accumulation of damaged protein, overwhelming cellular repair mechanisms and accelerating tissue dysfunction.

## 5. Conclusions

Although fried foods continue to be a popular choice because of their delicious flavors and textures, it is still difficult to guarantee their safety while maintaining their sensory appeal. This review has pointed out that the formation pathways of AA and AGEs are well-known, and they are often interlinked. Future studies should concentrate on improving understanding of the processes that result in the production of hazardous compounds under various frying applications, such as acrylamide and advanced glycation end products (AGEs). We therefore propose the possibility of innovative food processing techniques like steam frying, microwave-assisted frying, and pulsed electric field (PEF) treatment, which have demonstrated promise in lowering hazardous byproducts without affecting product quality. The impact of several bioactive compounds, including antioxidants, polyphenols, and fiber, in reducing the production of acrylamide and AGEs must be studied immediately in addition to optimizing frying parameters like temperature and time. To improve food safety without sacrificing the quality of fried foods, functional additives that can be added to food matrices to prevent or slow down certain chemical reactions may be developed. In addition, the food sector and regulatory agencies need to work together to develop more precise standards for the permissible amounts of acrylamide and AGEs in food products. This will encourage creative ways to reduce harmful substances while also helping in the development of safety standards. Consumer education about the risks of consuming excessively fried food and the health advantages of cooking methods that minimize harmful compounds should also be the focus of research. Raising awareness through public health campaigns may be essential to reducing exposure to these harmful drugs while improving fundamental nutrition. In conclusion, mitigating AA and AGEs is a complex challenge that requires an integrated approach, and more comprehensive research is required to find workable solutions for reducing their amount in fried food. A complete strategy that incorporates bioactive ingredients, process optimization, and consumer education will be essential to preventing the possible hazards of fried food from outweighing its advantages.

## Figures and Tables

**Table 1:** Acrylamide content in various food products, including potato samples with specific treatments.

Food Product	Cooking Method	Acrylamide Contents	Risk Category ^a^	Detection Method	Reference
Potatoes chips	Vaccum-treated	1166 μg/kg	High	HPLC-MS	[27]
Cookies	Vaccum-treated	366 μg/kg	Moderate	HPLC-MS	[27]
Fried potatoes	Frying	2022 μg/kg	High	GC-MS	[28]
Potatoe tubers (W)	Frying	1292 μg/kg	High	HPLC-MS	[26]
Potatoe tubers (PW)	Frying	886 μg/kg	Moderate	HPLC-MS	[26]
Potatoe tubers (Y)	Frying	1375 μg/kg	High	HPLC-MS	[26]
French fries	Deep-frying	2089 μg/kg	High	HPLC-UV	[29]
Hamburger	Frying	14 μg/kg	Low	GC-MS	[30]
Potato crips	Frying	1538 μg/kg	High	GC-MS	[30]
Bread	Oven-baked	49 μg/kg	Low	LC-MS	[30]
Crips bread	Oven-baked	1731 μg/kg	High	LC-MS	[30]
Roasted coffee	Roasting	203 μg/kg	Low	HPLC-MS	[31]
Instant coffee	Roasting	620 μg/kg	Low	HPLC-MS	[31]
Beef	Frying	20 μg/kg	Low	GC-MS	[30]
Pork	Frying	52 μg/kg	Low	GC-MS	[30]

W: Potato tubers dipped in water. PW: Potato tubers treated with pulsed electric field in water. Y: Potato tubers dipped in yeast aqueous suspension. ^a^ Risk categories are adopted from EU Commission Regulation 2017/2158 [32].

**Table 2:** AGEs contents in different foods.

Food Products	Cooking Method	AGE Contents	Marker	Risk Category ^a^	Detection Method	Reference
Bread and biscuits	Baking	178.36 μg/kg	CML	Low	UPLC-MS	[44]
Cereals	Roasting	281.29 μg/kg	CML	Moderate	UPLC-MS	[44]
Potatoes	Frying	25.16 μg/kg	CML	Moderate	UPLC-MS	[44]
Coffee	Roasting	84.12 μg/kg	CML	High	UPLC-MS	[44]
Chicken breast	Fried	23.54 μg/kg	CML	Moderate	UPLC-MS	[44]
Milk (fat free)	pasteurized	1 KU/100 g	CML	Low	ELISA	[15]
Milk (Whole)	Pasteurized	5 KU/100 g	CML	Low	ELISA	[15]
Fried pork	Frying	17.53 μg/kg	CML	High	HPLC-FLD	[45]
Baked salmon	Baking	8.59 μg/kg	CML	Moderate	HPLC-FLD	[45]
Apple	-	45 μg/kg	CML	High	ELISA	[15]
Banana	-	9 μg/kg	CML	Low	ELISA	[15]
Tomato	-	11 μg/kg	CML	Moderate	ELISA	[15]
Green beans, canned	-	18 μg/kg	CML	Moderate	ELISA	[15]
Soup and sauces	Boiling	24.33 μg/kg	CML	Low	UPLC-MS	[44]

^a^ Risk category is from the EU Commission Regulation 2017/2158 [32].

## Data Availability

No new data were created or analyzed in this study. Data sharing is not applicable to this article.

## Abbreviations

Acrylamide (AA); advanced glycation end products (AGEs); Amadori products (ARPs); Nε-(carboxymethyl)- lysine (CML); epidermal growth factor receptor protein (EGFR); N-(carboxyethyl) lysine (CEL); ultra-high pressure (UHP); glycidamide (GA); methyl glyoxal (MGO); reactive oxygen species (ROS); reactive carbonyl compounds (RCO); dibutylhydroxytoluene (BHT); glyoxal (GO); receptor of advanced glycation end products (RAGEs); (5-HMF) 5-hydroxymethylfurfural; vascular endothelial growth factor receptor 2 (VEGFR2); dietary advanced glycation end products (dAGEs); single-nucleotide polymorphism (SNP); International Agency for Research on Cancer (IARC); concanavalin A (ConA); (mitochondrial DNA) mtDNA; the Joint Expert Committee on Food Additives (JECFA); European Union’s Commission Regulation (EU); malondialdehyde (MDA); chronic kidney disease (CKD); (phosphoinositide 3-kinases) PI3K; (protein kinase B) AKT; (mammalian target of rapamycin) mTOR; brain-derived neurotrophic factor (BDNF); interleukin-6 (IL-6), signal transduction transcriptional activator 3 (STAT3); and hypoxia-inducible factor (HIF-1α).
